# *Toxoplasma gondii* and* Neospora caninum* in Free-Range Chickens in Henan Province of China

**DOI:** 10.1155/2016/8290536

**Published:** 2016-05-05

**Authors:** Yongjie Feng, Yaoyao Lu, Yinghua Wang, Jing Liu, Longxian Zhang, Yurong Yang

**Affiliations:** ^1^College of Animal Science and Veterinary Medicine, Henan Agricultural University, Zhengzhou 450002, China; ^2^Henan Province Center for Animal Disease Control and Prevention, Zhengzhou 450002, China; ^3^College of Veterinary Medicine, China Agricultural University, Beijing 100094, China

## Abstract

*Background.* Chickens serve as an intermediate host for* Toxoplasma gondii* and* Neospora caninum*; infection of free-range (FR) chickens with these organisms is a useful indicator of soil and environmental contamination with oocysts. A total of 700 FR chicken serum samples and 300 heart samples were collected from Henan province from March to July 2015. Antibodies to* T. gondii *were found in 18.86% (132/700) of the chickens by modified agglutination test (cut-off 1 : 25), while 23.14% (162/700) were positive for* N. caninum *by indirect fluorescent antibody test (cut-off 1 : 25).* T. gondii *DNA was detected in the myocardium digestion liquids of 4/25 (16%) FR chickens. The PCR results of* N. caninum* DNA from FR chicken myocardium digestion liquids (*n* = 25) were all negative. Attempts to isolate viable* T. gondii* were unsuccessful. The results showed that there were antibodies to* T. gondii* and* N. caninum* in FR chickens from Henan province. Accordingly, effective control of feces from cats and dogs and improved pets hygiene habits were needed. To the author's knowledge, this is the first report of the presence of* N. caninum* antibody in chickens from China.

## 1. Background

Many studies have evaluated* Toxoplasma gondii *and* Neospora caninum *infections in birds worldwide, including chickens [1, 2]. Chickens contribute to the* T. gondii* and* N. caninum* life cycle by acting as intermediate hosts or mechanical vectors [3, 4]. The main route of infection for chickens is assumed to be through ingestion of oocysts from soil. Limited reports of natural neosporosis in birds indicate that they do not show any significant histological lesions [4, 5]. Chickens are resistant to toxoplasmosis, and most* T. gondii* positive chickens remain asymptomatic. However, investigation of* T. gondii* and* N. caninum* infection in the free-range (FR) chickens is a useful indicator for assessment of soil and environmental contamination with oocysts.

Despite the high body temperature of avians (41°C), they could be infected by toxoplasmosis. Further,* T. gondii* tachyzoites have been observed in avian red blood cells [1]. Meat from chickens is consumed widely and is known to be the primary source of toxoplasmosis infection for humans. Viable* T. gondii* have been widely isolated from chicken heart, brain, and muscle in North America and Europe by bioassay in mice or cats [2]. China has the highest number of chickens in the world (approximately 4 billion, https://top5ofanything.com/list/0cffba91/Countries-With-the-Most-Chickens) and an unknown number of wild birds. However, only two viable* T. gondii* have been isolated from chickens in China [6, 7]. Reports of the genotype and pathogenicity of* T. gondii* revealed significant differences among populations in Asia and other parts of the world [2, 8]. Accordingly, further studies to develop methods of isolating* T. gondii* from China are warranted.

Few English papers have investigated the seroprevalence of* T. gondii* in birds from China. Yan et al. summarized the literature (1985–2008) pertaining to* T. gondii *prevalence in chickens from China [9]. Although chickens, sparrows, and pigeons have been shown to be intermediate hosts of* N. caninum* [4], there is little information regarding the* N. caninum* seroprevalence in chickens from China. The present study was conducted to investigate the prevalence of* N. caninum *and* T. gondii* infections in FR chickens from central China and to isolate* T. gondii*.

## 2. Methods

### 2.1. Sera Samples

**Figure 1:**
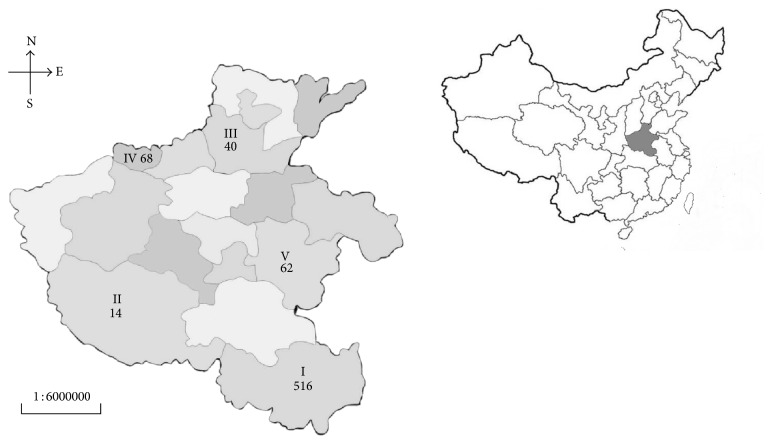
Location and number of samples received from Henan province of China. I: Xinyang; II: Nanyang; III: Xinxiang; IV: Jiyuan; V: Zhoukou.

The study area was Henan province (33.54°N, 113.30°E), which is located in central China. A total of 700 FR chicken sera and 300 fresh hearts from individual farm in Henan province were collected (Figure 1 and Table 1). Clinical information (age, gender) regarding all chickens except batch 5 and batch 6 was obtained.

### 2.2. Examination of Chickens Sera for* T. gondii* and* N. caninum* Antibodies

Sera samples from 700 FR chickens were tested for antibodies to* T. gondii* using the modified agglutination test (MAT) [2].* T. gondii* positive serum and* N. caninum* positive serum from mice were provided by Dr. J. P. Dubey (ARS, USDA) as reference sera. Whole formalin-treated* T. gondii* tachyzoites antigens were obtained from Kerafast Company (catalog number EH2002). Additionally, 700 serum samples were tested for* N. caninum* antibodies by the indirect fluorescent antibody test (IFAT). The 96-well IFAT plates (whole formalin fixed NC1* N. caninum* tachyzoites) were kindly provided by Dr. Q. Liu (China Agricultural University, China). The IFAT secondary antibody was goat anti-chicken IgY H&L labeled with Alexa Fluor 488 (Abcam Company, ab150169). Positive control, negative control, and blank were performed on each plate. A titer of 1 : 25 was considered to indicate exposure to* T. gondii *in chickens [2, 3]. In addition, all serum samples were tested for* T. gondii* parasites at 1 : 50, 1 : 100, and 1 : 200 dilutions. IFAT was used to test chicken serum* N. caninum* antibody (cut-off: 1 : 25), and only bright fluorescence of the whole tachyzoite surface was considered as a positive result [10].

### 2.3. Isolation of Viable* T. gondii* from Chicken Tissues by Bioassay of* Kunming* Mice

Chicken myocardia (10 g) from the samples with MAT of* T. gondii* seropositive (MAT, ≥50) chickens (*n* = 25) were digested individually in pepsin and bioassayed in* Kunming *(KM) outbred mice as previously described [2]. Each heart homogenate was inoculated subcutaneously into two mice. All inoculated mice were observed daily for illness. Dead mice were examined for* T. gondii* by making impression smears from the lung or brain, which were inoculated into a new group of mice regardless of whether they were negative or positive. Survivors were bled on 42 days postinoculation (DPI) and 1 : 25, 1 : 200 dilution of serum from the mouse was tested for* T. gondii* antibodies using the MAT. Mice were killed after 49 DPI, and brains of all mice were examined for tissue cysts and inoculated into new groups of mice. The sheep hearts containing* T. gondii *cysts were used as protocol control.

### 2.4. DNA Isolation and Polymerase Chain Reaction (PCR) Identification of* T. gondii* and* N. caninum*

Chickens myocardium digestion liquids from chicken hearts were also used to detect* T. gondii* DNA. The DNA isolated from* T. gondii* (CT1 strain) or* N. caninum* (NC1 strain) was used as a reference for PCR. CT1 strain* T. gondii* was kindly provided by Dr. J. P. Dubey (ARS, USDA). NC1 strain* N. caninum *was kindly provided by Dr. Q. Liu (China Agricultural University, China). The DNA of digestion heart samples was extracted using a commercial DNA extraction kit (Tiangen Biotec Company, DP304). PCR assays for* T. gondii* and* N. caninum *were performed using the specific primer pairs TOX5/TOX8 (5′-CGCTGCAGACACAGTGCATCTGGATT-3′ and 5′-CCCAGCTGCGTCTGTCGGGAT-3′) and NP6/NP21 (5′-CAGTCAACCTACGTCTTCT-3′ and 5′-GTGCGTCCAATCCTGTAAC-3′) [11, 12]. The products from* T. gondii *were expected to be 450 bp.* N. caninum *expected products were 328 bp. Briefly,* T. gondii* reaction cycle was as follows: initial denaturation at 94°C for 2 min, followed by 35 cycles of amplification (94°C for 1 min, 60°C for 1 min, and 72°C for 1 min) and then final extension at 72°C for 10 min.* N. caninum *reaction cycle was as follows: initial denaturation at 94°C for 3 min, followed by 35 cycles of amplification (94°C for 1 min, 53°C for 1 min, and 72°C for 2 min) and then final extension at 72°C for 10 min.

### 2.5. Statistical Analysis

Statistical analysis was performed by GraphPad Prism 4.0 software (GraphPad Software Inc., San Diego, CA, USA). Data were analyzed by the Chi-square test or Fisher's exact test to determine the association between infection with each parasite and independent risk factors: gender (male and female) and age (≤30 days, >30 days). *p* < 0.05 was considered statistically significant.

## 3. Results and Discussion

Antibodies to* T. gondii *were found in 18.86% (132/700) of FR chickens from Henan province of China (Table 1). Seropositivity rates varied with respect to gender, with antibodies being found in 33.59% of males and 14.48% of females (*p* < 0.01) (Tables 2 and 3). This was probably due to a greater probability of exposure to* T. gondii* oocysts in males because of the active characteristic of roosters. Many studies indicated that gender was not a risk factor for toxoplasmosis [1, 13]. However,* T. gondii* change the concentration of steroid hormones in the host and enhance the susceptibility of males or females to toxoplasmosis; it was decided by the stains* T. gondii* [14, 15]. A high percentage of male sheep were also positive for* T. gondii* antibody based on serological survey of China (*p* > 0.05) (Feng et al., unpublished data).

The seroprevalence of* T. gondii* is age related, with higher seroprevalence being observed in older chickens (>30 days, 18.75%) than in younger chickens (≤30 days, 15.85%); however, this difference was not significant (Tables 2 and 3). It is worth noting that 15.85% of 82 young chickens were positive for* T. gondii *antibody, indicating maybe vertical transmission from embryos.

Among the 700 FR chickens tested for* N. caninum* antibodies, 23.14% (162/700) were positive (Tables 2 and 3). The PCR results of* N. caninum* DNA from FR chicken myocardium digestion liquids (*n* = 25) were all negative. Age and gender showed no association with seroprevalence (*p* > 0.05). Additionally, 3.57% (25/700) FR chickens mix was exposed to the* T. gondii* and* N. caninum*. Moreover, 25.61% (21/82) of younger chickens (<30 days) were seropositive for* N. caninum*. An epidemiological survey of cattle and dogs supported the postnatal transmission of* N. caninum *[16, 17]. The high seropositive rate in younger chickens indicated that* N. caninum* transmission possibly occurs via eggs in birds. However, vertical transmission by* N. caninum* in chickens was not detected by microscopy, PCR, or BALB/c mice bioassays [18]. The methods of microscopy and PCR were specific and sensitive, but there were limitations for only checking few samples. It was temporal tissue distribution and parasite loads during* N. caninum* infection in BALB/c murine model [19]. Gerbils, immunodeficient mice, and cell cultures were the successful model for isolating* N. caninum* from tissues [20]. The exact evidence for* N. caninum* infection from hen to egg needs to be further investigated and proved.

*T. gondii* was not isolated from the mice inoculated with tissues of any chickens hearts in this study. The sheep hearts containing* T. gondii *cysts were used as protocol control. This excludes the errors of the protocol, such as pH of the solutions, enzymatic activity, and period of digestion. It has been shown that the density of* T. gondii* cysts in hearts is higher than in brain or muscle in chicken, and the heart is the ideal choice for isolation of* T. gondii* [2, 21].* T. gondii* bioassays in cats are more sensitive than in mice. The use of gamma interferon gene knockout mice or immunosuppressed mice facilitates early detection of* T. gondii* because most strains of* T. gondii* are asymptomatic in outbred mice. Sreekumar did not isolate any viable* T. gondii* from 186 (133 MAT seropositive) chicken tissues from India by mouse bioassay [22]. However, Qian successfully isolated* T. gondii* (11 isolations from 23 cats tissues) using immunosuppressed* Kunming* mice [23].* T. gondii* could grow efficiently in macrophages of rats treated with glucocorticoide [24]. The unsuccessful isolation may be related to the low density of* T. gondii *cysts burden in chickens of different regions or the natural resistance to* T. gondii* infection in some strains of mice. PCR indicated that the presence of* T. gondii* DNA was 16% (4/25) in FR chicken myocardium digestion liquids, which yielded PCR product of approximately 450 bp. The low infection rate observed upon molecular analysis verified the light burden of* T. gondii* cysts in chickens from central China.

## 4. Conclusions

Our results showed that there were antibodies to* T. gondii* and* N. caninum* in FR chickens from Henan province of China. The contamination environment was the source of infection with toxoplasmosis for humans and other animals. Accordingly, effective control of feces from cats and dogs and improved pets hygiene habits were needed.

## Figures and Tables

**Table 1:** Prevalence of *T. gondii* in free-range chickens from Henan of China.

Batch number	Location^d^	City/sample reception date	Number of samples received	Number of seropositive samples (cut-off: 1 : 25, %)	Isolates obtained by mice from hearts^a^
1	I	Xinyang/9 March 2015	201^b^	56 (27.9)	—
2	III	Xinxiang/9 March 2015	40	3 (7.5)	—
3	IV	Jiyuan/9 March 2015	68	10 (14.7)	—
4	II	Nanyang/9 March 2015	14	6 (42.9)	—
5	I	Xinyang/8 May 2015	7^c^	2 (28.6)	0/2
6	I	Xinyang/10 May 2015	51^c^	12 (23.5)	0/8
7	V	Zhoukou/14 May 2015	62	29 (46.8)	0/15
8	I	Xinyang/20 May 2015	13	0	—
9	I	Xinyang/24 May 2015	14	1 (7.1)	—
10	I	Xinyang/2 June 2015	15	0	—
11	I	Xinyang/9 June 2015	17	0	—
12	I	Xinyang/1 July 2015	7	0	—
13	I	Xinyang/8 July 2015	191^b^	13 (6.8)	—
Total			700	132 (18.86)	0/25

^a^Number of positive groups/number of inoculated groups.

^b^No heart tissue available.

^c^No detailed information regarding chickens.

^d^Sampling city in Figure 1.

**Table 2:** Seroprevalence of *T. gondii *and *N. caninum* infection in free-range chickens.

Characteristics	Number tested	Positive number in different titers				Seroprevalence % (positive number)	95% CI
		1 : 25	1 : 50	1 : 100	Above 1 : 200		
*T. gondii* (MAT, cut-off: 1 : 25)							
Gender: female	511	46	10	4	14	14.48 (74)	11.68–17.81
Gender: male	131	37	2	2	3	33.59 (44)	26.06–42.05
Age (days) ≤ 30	82	10	0	0	3	15.85 (13)	9.37–25.40
Age (days) > 30	560	73	12	6	14	18.75 (105)	15.73–22.20
No information^a^	58	9	0	0	5	24.14 (14)	14.85–36.64
Total	700	92	12	6	22	18.86 (132)	16.13–21.93

*N. caninum* (IFAT, cut-off: 1 : 25)							
Gender: female	511	123	—	—	—	24.07 (123)	20.56–27.97
Gender: male	131	27	—	—	—	20.61 (27)	14.52–28.38
Age (days) ≤ 30	82	21	—	—	—	25.61 (21)	17.34–36.06
Age (days) > 30	560	129	—	—	—	23.04 (129)	19.73–26.70
No information^a^	58	12	—	—	—	20.69 (12)	12.10–32.92
Total	700	162	—	—	—	23.14 (162)	20.17–26.41

^a^No detailed information (gender, age) regarding chickens.

**Table 3:** Odds ratio of gender and age of chickens as risk factors for *T. gondii *and *N. caninum*.

Factor	Category	OR	95% CI	*p* value
*T. gondii*				
Gender	Female	—	—	—
	Male	2.987	1.926–4.630	*p* < 0.0001^*∗*^
Age (days)	≤30	—	—	—
	>30	1.225	0.653–2.299	*p* = 0.6471

*N. caninum*				
Gender	Female	—	—	—
	Male	1.221	0.764–1.953	*p* = 0.4874
Age (days)	≤30	—	—	—
	>30	1.150	0.675–1.961	*p* = 0.5795

OR: odds ratio; “*∗*” indicates significant difference.
